# Complete Genome Sequence of the Type Strain Pectobacterium punjabense SS95, Isolated from a Potato Plant with Blackleg Symptoms

**DOI:** 10.1128/MRA.00420-20

**Published:** 2020-08-06

**Authors:** Sohaib Sarfraz, Saïd Oulghazi, Jérémy Cigna, Shahbaz Talib Sahi, Kashif Riaz, Muhammad Rizwan Tufail, Amna Fayyaz, Khalid Naveed, Akhtar Hameed, Céline Lopez-Roques, Céline Vandecasteele, Denis Faure

**Affiliations:** a Institute for Integrative Biology of the Cell (I2BC), CEA CNRS University Paris-Saclay, Gif-sur-Yvette, France; b Department of Plant Pathology, University of Agriculture Faisalabad, Sub-campus Depalpur, Okara, Pakistan; c Department of Plant Pathology, University of Agriculture Faisalabad, Faisalabad, Pakistan; d Department of Biology, Faculty of Sciences, Moulay Ismaïl University, Meknes, Morocco; e Fédération Nationale des Producteurs de Plants de Pomme de Terre (FN3PT), Paris, France; f INRA, US 1426, GeT-PlaGe, Genotoul, Castanet-Tolosan, France; Indiana University, Bloomington

## Abstract

Pectobacterium punjabense is a newly described species causing blackleg disease in potato plants. Therefore, by the combination of long (Oxford Nanopore Technologies, MinION) and short (Illumina MiSeq) reads, we sequenced the complete genome of *P. punjabense* SS95^T^, which contains a circular chromosome of 4.793 Mb with a GC content of 50.7%.

## ANNOUNCEMENT

The family *Pectobacteriaceae* encompasses pectinolytic plant pathogens that represent a threat to economically important vegetable crops and ornamental plants. *Pectobacterium* spp. are responsible for rotting diseases such as carrot or melon soft rot and potato blackleg and soft rot (1). Over the past decade, advances in genomics have allowed the scientific community to clarify the taxonomic position of many *Pectobacterium* species by either reexamining biological resources in the international collections or sampling a wider range of environments, from plants to surface waters (2–4). The type strain Pectobacterium punjabense SS95 (CFBP 8604, LMG 30622) was isolated from potato plants showing blackleg symptoms collected from Punjab, Pakistan, in 2017 (5). Serially diluted samples were plated onto crystal violet pectate (CVP) agar medium, and plates were incubated for 48 h at 28°C (6). Bacterial colonies producing pitting on CVP were purified on nutrient agar (beef extract [3 g], peptone [5 g], glucose [2.5 g], and agar [15 g per liter]). Genomic DNA was extracted using a MasterPure complete DNA purification kit (Epicentre, Madison, WI, USA). DNA quantification and quality control were performed using a Qubit 2.0 fluorometer and 1.0% agarose gel electrophoresis. Whole-genome shotgun DNA sequencing of *P. punjabense* SS95 was performed by a combination of Illumina MiSeq and Oxford Nanopore Technologies (ONT) MinION sequencing. The library was prepared using the Nextera DNA Flex kit (Illumina), and sequencing was performed using the MiSeq reagent kit v.2 with paired-end chemistry (2 × 150 bp). The sequence reads were trimmed to remove adapter sequences with Cutadapt v.1.15, and quality control was performed using FastQC v.0.11.5 (http://www.bioinformatics.babraham.ac.uk/projects/fastqc).

The ONT library preparation and sequencing were performed at the GeT-PlaGe core facility (INRA, Toulouse), according to the manufacturer’s instructions, by following the 1D native genomic DNA barcoding protocol (EXP-NBD103 and SQK-LSK108). At each step, the DNA was quantified using the Qubit double-stranded DNA (dsDNA) high-sensitivity (HS) assay kit (Life Technologies). The DNA purity was tested using the NanoDrop spectrophotometer (Thermo Fisher), and the size distribution and degradation were assessed using the Fragment Analyzer high-sensitivity DNA fragment analysis kit (AATI). Purification steps were performed using AMPure XP beads (Beckman Coulter). Using the Megaruptor 1 system (Diagenode), 5 μg of DNA was sheared at 20 kb. A DNA damage repair step was performed on 3 μg of sample. Then, end repair and dA tailing of double-stranded DNA fragments were performed on 1 μg of sample. The library was generated; then, adapters were ligated onto the library, and it was loaded onto an R9.4.1 flow cell and sequenced on a MinION instrument at 0.15 pmol within 48 h. The raw sequence data (fast5 format) from ONT sequencing were obtained with MinKNOW v.1.10.23 and were base called with ONT Albacore Sequencing Pipeline Software v.2.1.10; reads passing the internal test were used for the subsequent analysis. Porechop v.0.2.1 (https://github.com/rrwick/Porechop) was used for adaptor trimming. After filtering (quality, >9; length, >3,000 nucleotides), the ONT data used for the assembly showed an average length of 10,431 bp and an *N*_50_ value of 11,971 bp. The 71,148 ONT reads were assembled using Canu v.1.7 (7) with the “genomeSize=5m” and “minReadLength=3000” options (genome coverage, 155×). The 1,144,952 Illumina reads were mapped onto the ONT assembly with Burrows-Wheeler Aligner MEM v.0.7.12 (8) for sequence and assembly error correction with Pilon v.1.22 (9). The contig was finally circularized using Circlator v.1.5.1 (https://github.com/sanger-pathogens/circlator). The resulting sequence was annotated using the Prokaryotic Genome Annotation Pipeline (PGAP) (10), which predicted 4,307 coding sequences. The total genome size is 4,793,778 bp with a GC content of 50.7%.

The complete genome sequence of *P. punjabense* SS95^T^ provides essential data for studying its genetic diversity and host range, as well as comparative genomic analyses between its closest relative species.

### Data availability.

The Illumina (accession number SRR11674121) and MinION (accession number SRR11788435) reads were deposited in the Sequence Read Archive (SRA). The complete genome sequence of *Pectobacterium punjabense* SS95 was deposited under GenBank accession number CP038498.1.
